# Effect of Cisplatin and Its Cationic Analogues in the Phase Behavior and Permeability of Model Lipid Bilayers

**DOI:** 10.1021/acs.molpharmaceut.2c00321

**Published:** 2023-01-26

**Authors:** Nuno Martinho, Joaquim M. T. Marquês, Iryna Todoriko, Manuel Prieto, Rodrigo F.M. de Almeida, Liana C. Silva

**Affiliations:** † Research Institute for Medicines (iMed.ULisboa), Faculdade de Farmácia, Universidade de Lisboa, 1649-003Lisboa, Portugal; ‡ iBB—Institute for Bioengineering and Biosciences and Department of Bioengineering, Instituto Superior Técnico, Universidade de Lisboa, Av. Rovisco Pais, 1649-003Lisboa, Portugal; § Associate Laboratory i4HB—Institute for Health and Bioeconomy at Instituto Superior Técnico, Universidade de Lisboa, Av. Rovisco Pais, 1649-003Lisboa, Portugal; ∥ Centro de Química Estrutural, Institute of Molecular Sciences, Departamento de Química e Bioquímica, Faculdade de Ciências, Universidade de Lisboa, Campo Grande, 1649-003Lisboa, Portugal

**Keywords:** cisplatin, aquated cisplatin, membrane fluidity and permeability, gel phase, fluorescence spectroscopy

## Abstract

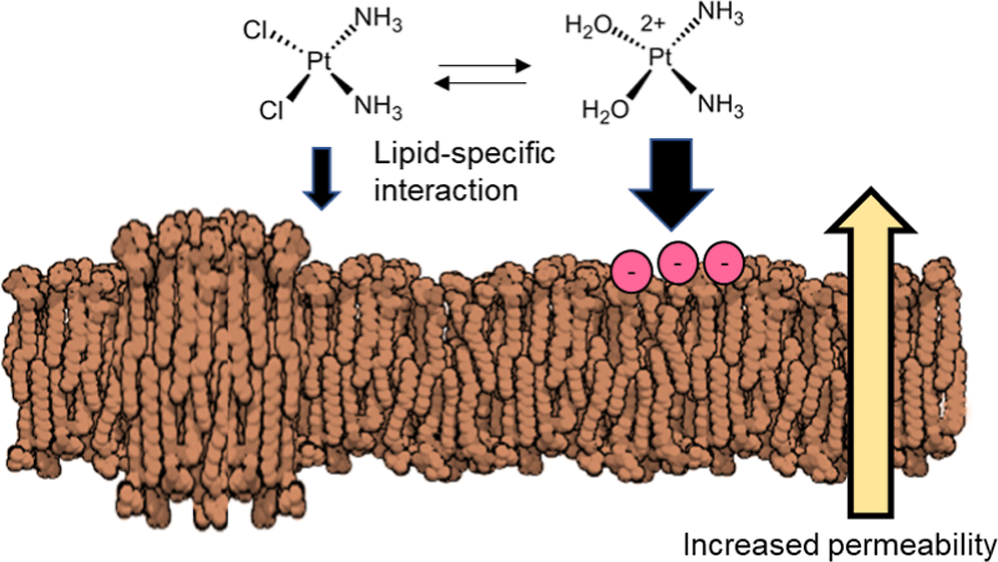

Increasing evidence suggests a critical role of lipids in both the mechanisms of toxicity and resistance of cells to platinum(II) complexes. In particular, cisplatin and other analogues were reported to interact with lipids and transiently promote lipid phase changes both in the bulk membranes and in specific membrane domains. However, these processes are complex and not fully understood. In this work, cisplatin and its cationic species formed at pH 7.4 in low chloride concentrations were tested for their ability to induce phase changes in model membranes with different lipid compositions. Fluorescent probes that partition to different lipid phases were used to report on the fluidity of the membrane, and a leakage assay was performed to evaluate the effect of cisplatin in the permeability of these vesicles. The results showed that platinum(II) complex effects on membrane fluidity depend on membrane lipid composition and properties, promoting a stronger decrease in the fluidity of membranes containing gel phase. Moreover, at high concentration, these complexes were prone to alter the permeability of lipid membranes without inducing their collapse or aggregation.

## Introduction

Cisplatin is a widely used anti-cancer drug in clinical practice for a variety of tumors.^1^ Inside cells, genomic DNA is reported to be the primary cellular target of platinum(II) complexes with which they form cross-linked adducts.^2^ However, growing evidence suggests that other non-DNA targets have an important role in cisplatin’s cytotoxicity.^3^ In fact, one major limitation associated with platinum(II) complexes is the mechanisms of resistance that are not dependent on DNA adduct formation, including a decrease of platinum accumulation inside the cell.^4−6^ These resistance mechanisms are multifactorial, and there are still many open questions regarding this issue.

Membrane lipids have shown to be involved in both cytotoxicity and resistance mechanisms. Before reaching its primary target, cisplatin has to cross the plasma membrane, which can occur via passive diffusion and receptor-mediated uptake.^3,7^ Although not yet fully understood, the passive diffusion depends on the physiological environment, including chloride concentration and pH, which can influence the formation of “aquated” platinum species (AqCis) in different percentages. These species have different physicochemical properties such as positive charge and thus interact differently with DNA (negatively charged), lipids, and lipid membranes.^8^ Moreover, the lipid membrane composition and permeability have been linked to the resistance mechanisms of cells to platinum(II) compounds,^4−6^ and membrane modulators, such as digitonin, were shown to increase the absorption of platinum complexes into cells.^9^ Cisplatin is also able to directly interact with phospholipid headgroup of several lipids and induce lipid phase changes, including non-lamellar phases (e.g., hexagonal II phase^10^). In turn, it has also been suggested that changing the curvature of the lipid bilayer may cause cisplatin to diffuse differently.^11^ Additionally, binding of platinum complexes to the headgroup of lipids induced structural changes at deeper locations of the lipid membrane,^7,12^ which were transient and recoverable within a few hours.^7,13^ Despite these interactions, whether lipid complexation is relevant for cisplatin’s mode of action is still yet to be determined.^13^

The molecular response regarding lipid metabolism has also been linked to the toxicity of cisplatin, and evidence suggests that activation of death signaling pathways might involve changes in lipid composition^14^ and lipid raft domains.^15^ Indeed, cisplatin was shown to induce ceramide formation in lipid rafts via acid sphingomyelinase (aSMase) activation,^16^ with a concomitant increase in membrane fluidity. However, membrane enrichment in ceramide commonly results in increased membrane order,^17,18^ suggesting that mechanisms other than ceramide formation might be responsible for the observed increased fluidity. This is also supported by observations that cisplatin (i) reduces the fluidity of erythrocytes and model membranes,^7^ (ii) increases the transition temperature and order of the aliphatic chains, and (iii) increases the thickness of the bilayer.^19^ Therefore, contradictory data are present in the literature regarding how cisplatin affects the biophysical properties of the membranes and whether these effects may be linked to its mechanisms of action.

To gain further insight into this subject, the impact of cisplatin and AqCis in the fluidity of lipid bilayers having different lipid compositions to mimic different membrane domains, and thus different biophysical properties, was evaluated, as well as its contribution to the permeability of these membranes. A better understanding of these factors should provide context regarding the lipid membrane interactions that lead to apoptosis and the potential mechanisms of resistance by changes in cell lipid composition.

## Materials and Methods

### Materials

POPC (1-palmitoyl-2-oleoyl-*sn*-glycero-3-phosphocholine), DPPC (1,2-dipalmitoyl-*sn*-glycero-3-phosphocholine), C16Cer (C16 ceramide, *N*-palmitoyl-d-*erythro*-sphingosine), C24:1Cer (C24:1 ceramide, *N*-nervonoyl-d-*erythro*-sphingosine), SM (sphingomyelin, from egg, chicken), and POPS (1-palmitoyl-2-oleoyl-*sn*-glycero-3-phospho-l-serine) were obtained from Avanti Polar Lipids, Inc. (Alabaster, AL, USA). DPH (1,6-diphenyl-1,3,4-hexatriene), TMA-DPH (1-(trimethylammonium)phenyl)-6-phenylhexa-1,3,5-triene), *t*-PnA (*trans*-parinaric acid), and CF (5(6)-carboxyfluorescein) were obtained from Molecular Probes/Invitrogen (Eugene, OR). Chol (cholesterol), cisplatin, and Triton X-100 (TX-100) were obtained from Sigma-Aldrich (St. Louis, MO, USA). All other chemicals were analytical grade from Merck and Fluka (St Louis, MO, USA) and were used without further purification.

The concentration of lipids in stock solutions was confirmed by phosphorus analysis.^20,21^ Chol and ceramide stock solution concentration were determined gravimetrically with a high-precision balance (Mettler Toledo UMT2, Columbus, OH). Probe concentrations were determined spectrophotometrically.^22^

### Large Unilamellar Vesicle Preparation

Multilamellar lipid vesicles (MLVs) were first prepared by a thin-film hydration freeze-thawing method. To this end, different aliquots of the desired lipid or lipid mixture [POPC, DPPC, POPC/DPPC (1:1), POPC/POPS (7:3), POPC/Chol (7:3), POPC/C24:1Cer (7:3), POPC/C16Cer (8:2), POPC/SM/Chol (1:1:1)] in chloroform were evaporated to dryness under a nitrogen stream with (DPH, TMA-DPH, CF) or without probes. Residual chloroform was further removed overnight under vacuum. The resultant lipid film was then hydrated with the appropriate buffer: (A) PBS: 10 mM Na_2_HPO_4_ with 150 mM of NaCl, pH 7.4; (B) PBS: 10 mM Na_2_HPO_4_ with 150 mM of Na_2_SO_3_, pH 7.4; or (C) 10 mM HEPES pH 7.4 with and without CF, used only for membrane permeability assays. For permeability assays, the osmolality was corrected to be similar between both buffers. The MLV suspension was then freeze-thawed for six cycles, and the resulting suspension was extruded 23 times through polycarbonate filters of 100 nm pore size (nucleopore, Pleasanton CA, USA) maintaining the temperature above the main transition temperature of lipids being used. In studies where *t*-PnA was used, the probe was added to the large unilamellar vesicle (LUV) suspension only after extrusion and incubated for at least 6 h.

### Dynamic Light Scattering and Electrophoretic Measurements

The average size (*Z*-average and number size) and zeta potential of LUV were measured on a ZetaSizer Nano ZS system (Malvern Instruments). The dynamic light scattering (DLS) measurements were carried out using a 90° scattering optics at 25 °C with parameters set for a viscosity of 0.890 cP and a refractive index of 1.330. All measurements were carried out using LUV at lipid concentrations between 0.1 and 1 mM.

The zeta potential (ζ) was determined from the electrophoretic mobility of LUV by means of the Helmholtz–Smoluchowski correlation. Measurements were carried out using disposable zeta potential cells at a concentration range of 0.1–0.5 mM, and the sample was maintained at 25 °C.

### Fluorescence Spectroscopy

Fluorescence anisotropy of DPH [lipid/probe ratio of 1:200], TMA-DPH (1:200), and *t*-PnA (1:500) in the different LUV suspensions (total lipid concentration of 1 mM) was measured on an SLM Aminco 8100 series 2 spectrofluorimeter. Measurements were carried out in a thermostated cell holder maintained at 25 °C using a Julabo F25 circulated water bath. Samples were placed in a quartz cuvette under magnetic stirring, and fluorescence anisotropy was measured (5 scans) with a slit bandwidth of 8 nm for both excitation and emission beams for DPH and TMA-DPH and 16 nm for *t*-PnA. The excitation (λ_exc_)/emission (λ_em_) wavelengths were set to 358/430 nm for DPH and TMA-DPH and to 305/405 nm for *t*-PnA. Platinum(II) complexes were then added to final concentrations of 15, 35, 100, and 300 μM. The steady-state fluorescence anisotropy was calculated from eq 1
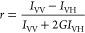
1where the intensities of the different polarized components of excitation and emission are described in subscript (V—vertical; H—horizontal).

### Electronic Absorption Spectroscopy

The absorption spectra of DPH, TMA-DPH, and *t*-PnA in LUV composed of different lipids were measured at different time points (0, 30 min, 4 h, 12 h, 24 h, 48 h, 72 h) in a Hitachi model U-2000 instrument in the 250–550 nm range using quartz cells with a 0.5 cm path length and a temperature of 25 °C. The derivative spectra were calculated using the Savitzky–Golay method in which a second-order polynomial convolution of 13 points was employed.^23^

### LUV Leakage Studies

LUVs at a lipid concentration of 2 mM were prepared as described above with 40 mM of CF in buffer C. The suspension containing LUV and non-encapsulated CF was then centrifuged (Ultracentrifuge Hitachi CP80NX with P70AT rotor) at 150 000 rcf for 2 h. The supernatant was removed, and the pellet was re-suspended in 1 mL of buffer and filtered through a Sephadex G-25 gel filtration column. The two 0.5 mL fractions where most of the LUVs were eluted, as verified by DLS and UV–vis spectrophotometry, were pooled. The recovered LUV suspension was then diluted in a 96-well plate to a final lipid concentration of 0.17–0.25 mM (a total volume of 250 μL). Cisplatin and AqCis were then added, and the fluorescence intensity read in a microplate reader SpectraMAX GeminiEM every 2 min at 25 °C for 14 h (λ_exc_/λ_em_ of 492/530 with a cutoff at 515 nm). Afterward, LUV lysis was promoted by addition of 10 μL of a 10% solution of TX-100 to each well and mixed gently for 30 min, after which 10 intensity reads were performed. The extent of CF release was determined as follows
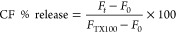
2where *F*_*t*_ represents the fluorescence intensity at time *t*, *F*_0_ represents the fluorescence intensity at the first measurement, and *F*_TX100_ represents the fluorescence intensity after addition of TX-100.

## Results

### Characterization of LUV by DLS

**Figure 1:**
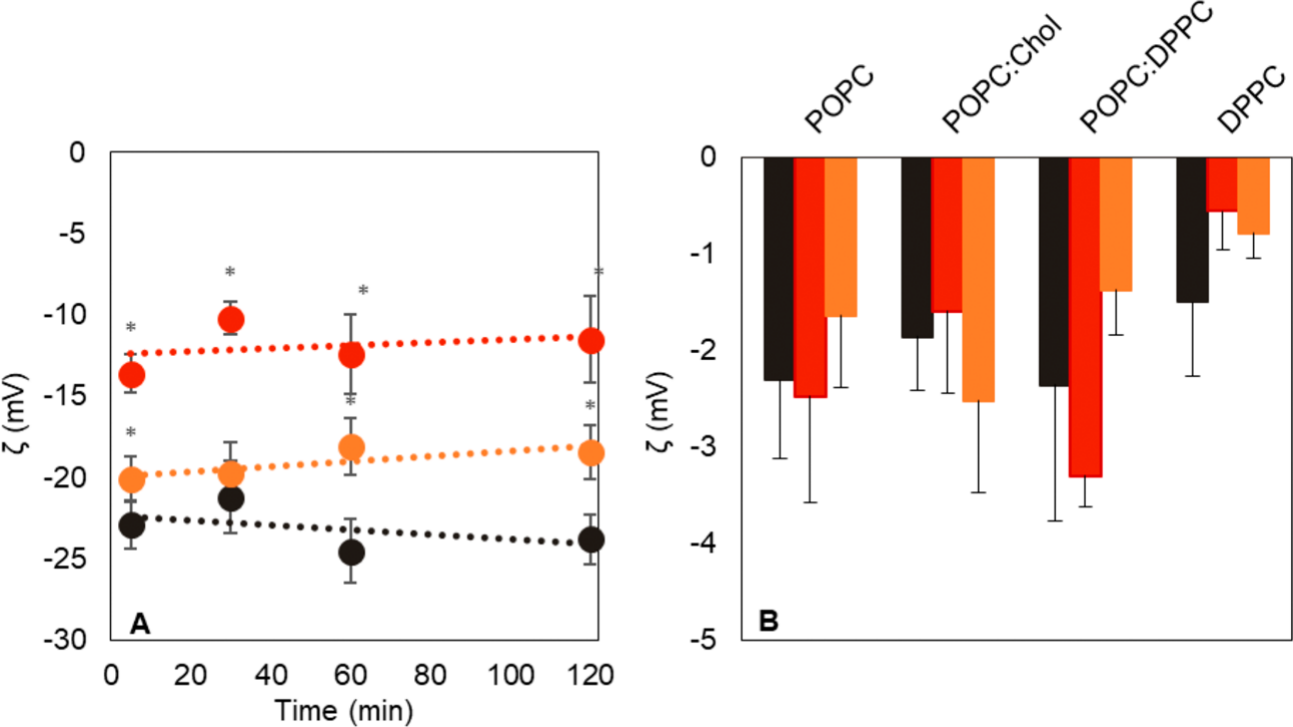
Effect of cisplatin and AqCis on the zeta potential of LUV with different lipid compositions. (A) Variation of the zeta potential of POPC/POPS (7:3) LUV in the absence (black line) and in the presence of AqCis at concentrations of 35 μM (orange line) and 300 μM (red line). (B) Effect of 300 μM of cisplatin (red bars) and AqCis (orange bars) on the zeta potential of LUV containing zwitterionic lipids (each control displayed as black bars) over a period of 24 h (ANOVA, *p < 0.05).

Cisplatin in aqueous solutions exists as an equilibrium of multiple neutral and charged species (p*K*_a_ values between 6.6 and 7.3), commonly named “aquated” species, which depends on both pH and concentration of chloride. At high chloride concentrations, cisplatin remains neutral, whereas at low chloride concentrations, it forms positively charged species. Therefore, the cationic AqCis was obtained in chloride-free buffers at pH 7.4, which according to previous reports^24^ should represent a mixture of 70% neutral species and 30% positively charged species. The formation of these charged species was verified indirectly by measurements of the zeta potential of POPC/POPS (7:3) LUV containing the negatively charged POPS lipid prepared in buffer with no chloride (Figure 1A). The results showed that the higher the concentration of AqCis, the higher the zeta potential of these vesicles due to membrane surface charge neutralization by cationic AqCis. Changes in zeta potential were not observed when increased concentrations of cisplatin were added in buffer with high chloride to POPC/POPS (7:3) LUV (−22.66 ± 0.98 and −23.16 ± 2.11 mV for 35 and 300 μM, respectively), confirming that surface charge neutralization was caused by AqCis cationic species. Furthermore, the zeta potential of POPC/POPS (7:3) prepared in high chloride in the presence of AqCis was similar to the control after 24 h, indicating a transient effect possibly due to reversal of AqCis back to cisplatin (data not shown). Finally, in zwitterionic models of POPC, POPC/DPPC (1:1), DPPC, and POPC/Chol (7:3) LUV, the presence of neither cisplatin nor AqCis significantly altered the zeta potential (Figure 1B).

The stability of the different LUVs (POPC, POPC/DPPC, DPPC, POPC/POPS, and POPC/Chol) in the presence of cisplatin or AqCis was also evaluated by measuring their average size after 24 and 48 h. No significant changes in size were observed in the presence of cisplatin or AqCis up to concentrations of 600 μM (data not shown), with *Z*_average_ sizes ranging from 88 to 150 nm. These results further indicate that the interaction of AqCis and cisplatin with the LUV did not induce aggregation and/or fusion of the vesicles.

### Effect of Platinum Complexes in the Fluidity of Different Phospholipid Mixtures

The fluidity of lipid membranes upon addition of platinum complexes was evaluated by measuring the fluorescence anisotropy of three different probes (DPH, TMA-DPH, and *t*-PnA). These probes were selected because they have different partition coefficients toward fluid or gel phases and, in addition, locate at different depths of the hydrophobic region, thus reporting the behavior of the lipid acyl chains at different depths of the bilayer.^22,25^ In this regard, both DPH and TMA-DPH partition equally between gel and fluid phases^18^ with DPH located near the center of the bilayer, whereas TMA-DPH is anchored at the surface and is more sensitive to hydration.^26^ On the other hand, *t*-PnA has a higher partition and quantum yield in the gel phase compared to liquid ordered and disordered phases.^18,27^

**Figure 2:**
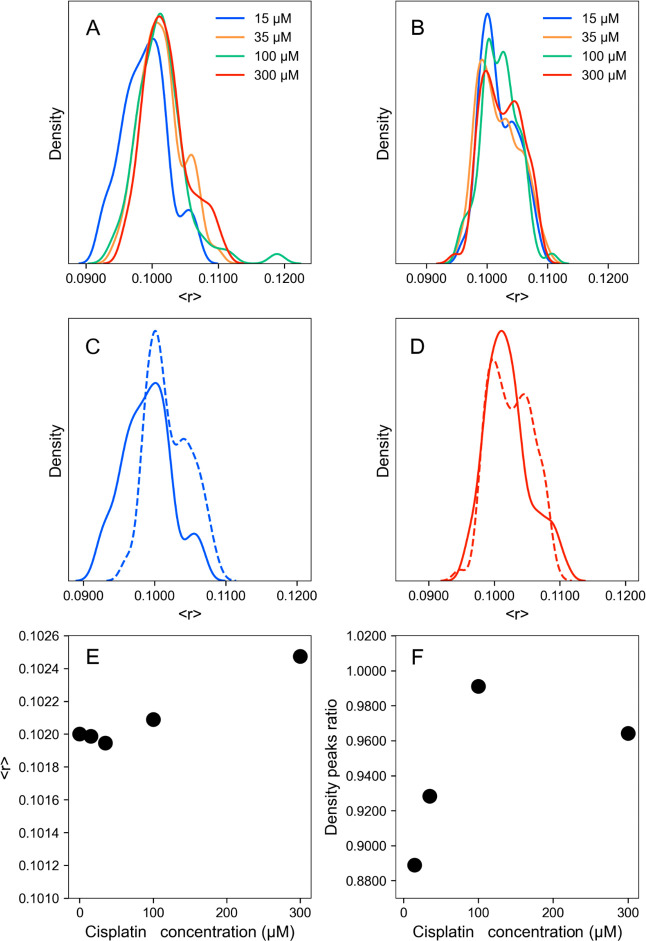
Effect of cisplatin concentration on the fluidity of POPC membranes. DPH fluorescence anisotropy density distribution (A) between 5 and 30 min (t1, 5 time points, 10 readings each, and 3 independent replicates) and (B) between 60 and 100 min (t2, 5 time points, 10 readings each, and 3 independent replicates) after adding 15 (blue), 35 (orange), 100 (green), and 300 μM (red) of cisplatin. Comparison between t1 (solid line) and t2 (dashed line) for POPC LUV in the presence of (C) 15 and (D) 300 μM cisplatin showed an increase in the contribution of a second peak centered at higher anisotropy at t2. (E) DPH anisotropy as a function of cisplatin concentration at t2. (F) Ratio between DPH anisotropy at the secondary peak over the primary peak (●). The average fluorescence anisotropy of DPH in POPC LUV in the absence of cisplatin (0.1020 ± 0.0035).

Time course measurements of DPH fluorescence anisotropy were performed on POPC LUV to address the effects of cisplatin and AqCis on membrane fluidity (Figure S1). A tendency for the appearance of higher anisotropy values could be noted at longer times and for higher cisplatin concentrations (Figure S1). However, the small absolute difference masked any clear trend. In this way, the data obtained within the first 30 min (5 time points, 10 readings each, and 3 independent replicates) and between 60 and 100 min (5 time points, 10 readings each, and 3 independent replicates) after addition of cisplatin were grouped in order to analyze the distribution of the fluorescence anisotropy measurements (Figure 2A,B). Within the first 30 min after cisplatin addition to POPC LUV, a normal distribution around the initial control values (⟨*r*⟩ of 0.1020 ± 0.0035) was observed for all concentrations tested, except for the lowest concentration of cisplatin (15 μM) where a slightly lower average anisotropy was obtained (⟨*r*⟩ of 0.1000 ± 0.0014), and the anisotropy profile distribution was shifted toward lower values. On the other hand, between 60 and 100 min, a very small concentration-dependent effect that resulted in increased anisotropy of DPH was observed, particularly for the highest cisplatin concentration (Figure 2B–F). In this time range, a small shift in the DPH anisotropy profile toward higher values was observed for the different concentrations tested, when compared to the first 30 min (Figure 2B–D). In this regard, DPH anisotropy distribution profile revealed the presence of two modes with anisotropy values centered at 0.1010 and 0.1050, and the contribution of the population with higher anisotropy increased over time for all cisplatin concentrations studied (Figure 2C–F). This increase in DPH anisotropy was not due a decrease in DPH fluorescence lifetime^28^ since no changes in DPH fluorescence lifetime were observed (τ ∼ 8.5 ns).^29^ These results suggest that cisplatin induces a very small and transient increase in the packing of the lipid acyl chains, possibly due to a slow penetration of the molecules to the interior of the bilayer and/or most likely a decrease in lipid–lipid headgroup distance due to increased electrostatic interactions promoted by cisplatin.

Contrary to cisplatin, increasing the concentrations of AqCis in the POPC LUV did not result in significant differences in DPH fluorescence anisotropy within the same timescale, even for the highest concentration (Figure S1). Differences were also not observed in the absorption spectra of DPH in the POPC LUV system at different time points upon addition of either cisplatin or AqCis (data not shown), which further evidenced the low influence of the platinum complexes on the biophysical properties of POPC bilayers.

Similarly, the effects of cisplatin and AqCis in a variety of LUVs with different lipid compositions [POPC/Chol (7:3), POPC/POPS (7:3), and POPC/DPPC (1:1)] also showed no significant differences in DPH anisotropy up to 3 h after addition of cisplatin or AqCis at different concentrations (Figure S2). Moreover, extending the interaction of cisplatin with these LUVs for 72 h did not show any significant alteration in fluorescence anisotropy as well as in the UV spectra (data not shown), suggesting that the effects of the platinum complexes in the fluidity of these membranes are negligible.

The interaction of cisplatin and AqCis with POPS-containing lipid systems and its possible effects on membrane fluidity were further characterized using *t*-PnA and TMA-DPH probes. No significant differences in *t*-PnA anisotropy were observed up to 24 h after addition of cisplatin (0.1426 ± 0.0049, for 300 μM) or AqCis (0.1414 ± 0.0075, for 300 μM) in buffer A (high chloride) to POPC/POPS (7:3) LUV compared to control (0.1399 ± 0.0063). In order to maintain the AqCis equilibrium and promote electrostatic interactions, measurements of the anisotropy of DPH and TMA-DPH in POPC/POPS (7:3) LUV were also carried out in buffer B (low chloride) to an extended period of 120 h. However, no differences in the fluorescence anisotropy of the probes could also be observed for neither cisplatin nor AqCis at the different concentrations tested when compared to the control (Figure S3 showcases a typical result for the highest concentration of 300 μM). These results suggest that both cisplatin and AqCis were unable to change the fluidity of this model membrane at different depths of the membrane despite the presumed initial electrostatic interaction between cationic AqCis and the POPS headgroup that led to surface charge neutralization (Figure 1).

**Figure 3:**
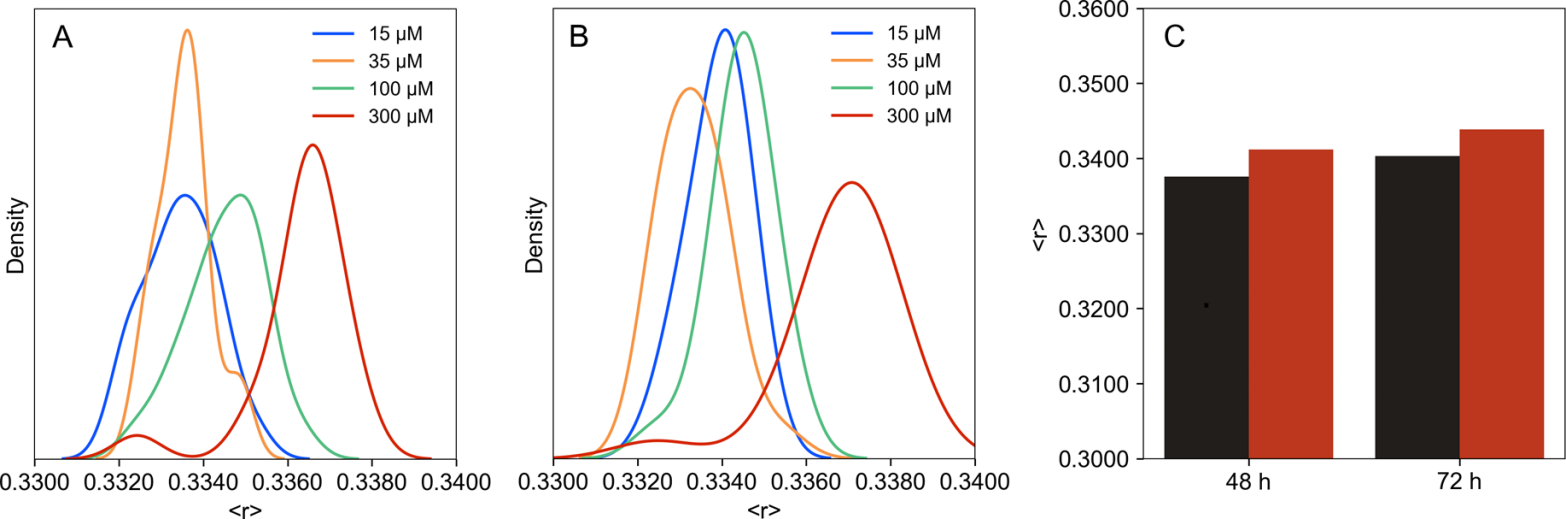
Effects of cisplatin and AqCis on the biophysical properties of gel phase DPPC model membranes. Fluorescence anisotropy distribution of DPH in DPPC LUV between 5 min and 3 h after addition of 15 (blue), 35 (orange), 100 (green), and 300 (red) μM of (A) cisplatin and (B) AqCis. The ordering effects of cisplatin were observed throughout 72 h (C) after addition of 300 μM of cisplatin (red bar) compared to the control (black). In (A,B), data correspond to the distribution of 15 time points, 10 readings each from 3 independent replicates; in (C), data correspond to the average ± SD of three independent experiments.

To further determine if cisplatin effects were dependent on membrane phase properties, the interaction of both complexes was studied in gel-phase DPPC vesicles (Figures 3 and S4). Contrary to the other lipid models, both cisplatin and AqCis induced a small decrease in membrane fluidity as observed by the increase of DPH anisotropy, particularly at the highest concentrations. This concentration-dependent effect was sustained and was observed even 72 h after addition of cisplatin (Figure 3C). On the other hand, no differences were observed in TMA-DPH anisotropy between the control (0.3185 ± 0.0023) and 120 h after adding cisplatin (0.3159 ± 0.0023) and AqCis (0.3160 ± 0.0013) to DPPC LUV.

### Influence of Cisplatin and AqCis on the Biophysical Properties of Sphingolipid-Containing Mixtures

**Figure 4:**
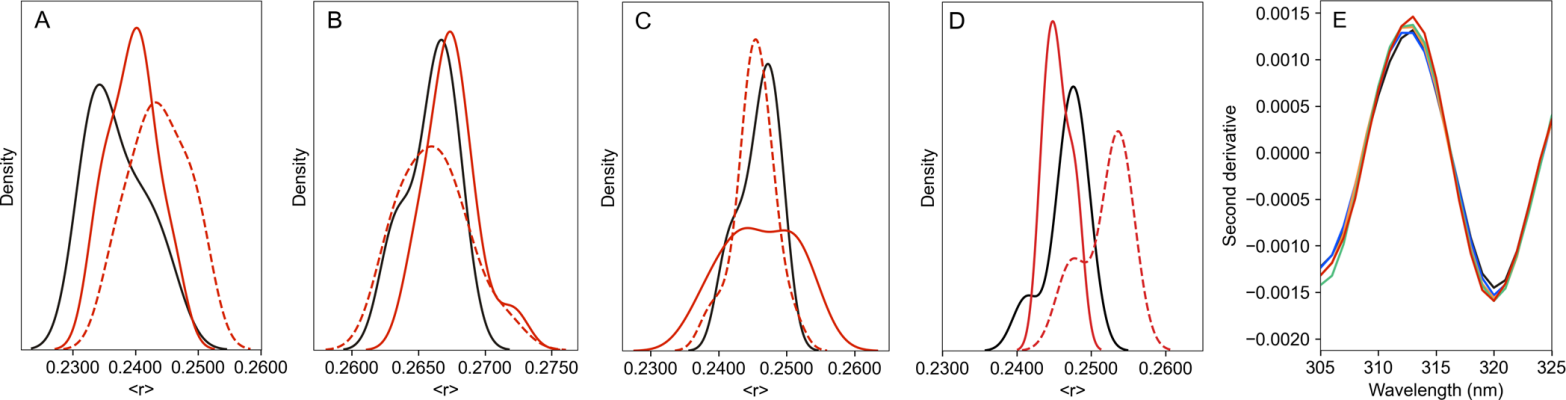
Effect of cisplatin and AqCis on the biophysical properties of sphingolipid-containing model membranes. Fluorescence anisotropy of *t*-PnA in (A) POPC/SM/Chol (1:1:1), (B) POPC/C16Cer (8:2), and (C) POPC/C24:1 (7:3) over 3 h after addition of 300 μM of cisplatin (red solid line) and AqCis (red dashed line); control is shown in black. (D) Fluorescence anisotropy of *t*-PnA in POPC/C24:1 (7:3) 30 min after addition of 300 μM of cisplatin (red solid line) and AqCis (red dashed line); control is shown in black. (E) Second derivative of the UV spectra of *t*-PnA in POPC/C24:1Cer LUV in the absence (gray) and 30 min after addition of 15 (blue), 35 (orange), 100 (green), and 300 (red) μM of cisplatin where a small concentration-dependent effect was observed.

**Figure 5:**
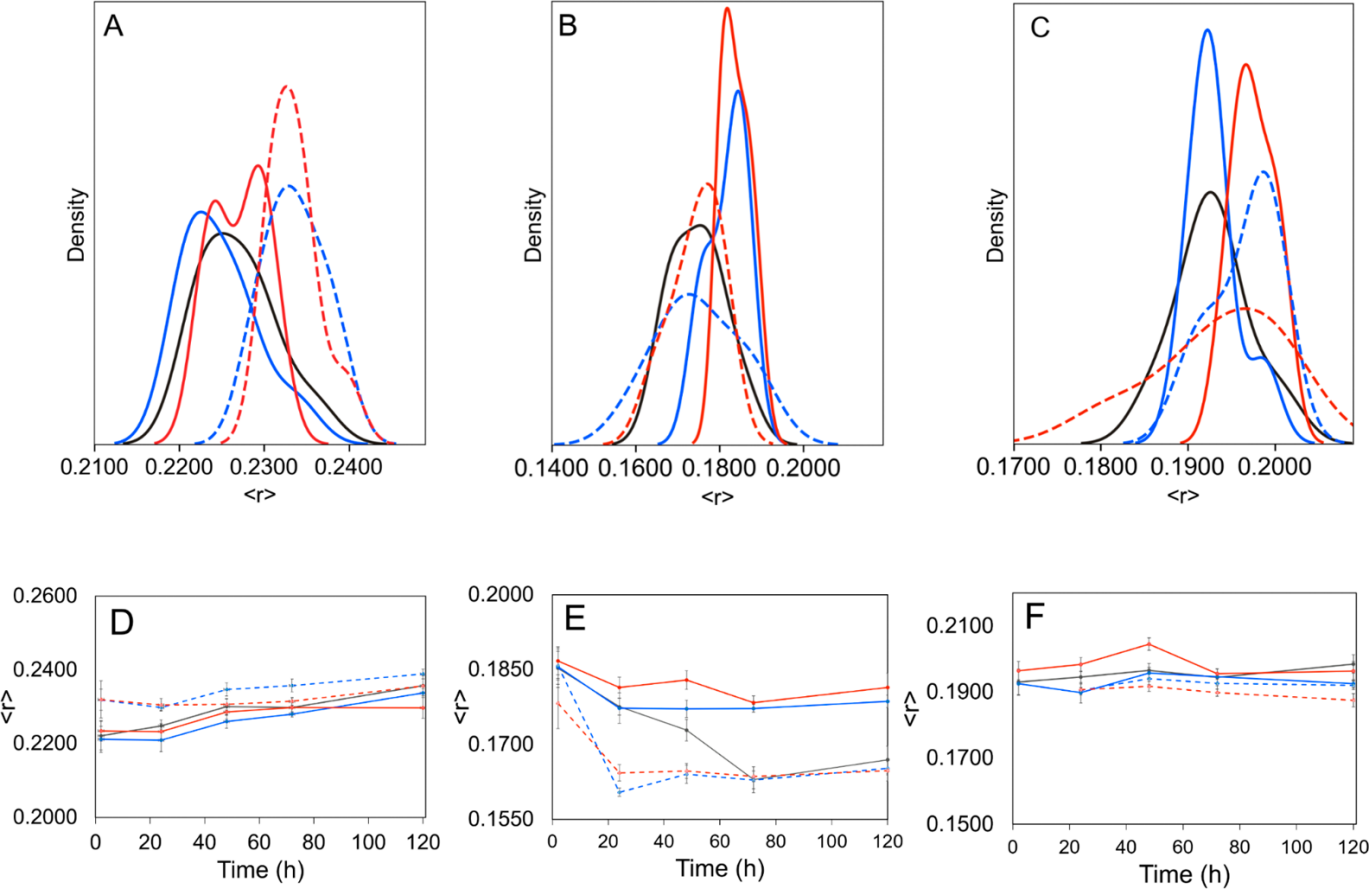
Effect of cisplatin and AqCis on the biophysical properties of sphingolipid-containing model membranes. Fluorescence anisotropy of TMA-DPH in (A,D) POPC/SM/Chol (1:1:1), (B,E) POPC:C16Cer (8:2), and (C,F) POPC/C24:1Cer (7:3) after addition of 15 μM (blue) and 300 μM (red) of cisplatin (solid line) or AqCis (dashed line); control (black); (A–C) represents the distribution of anisotropy values up to 2 h.

The probes *t*-PnA and TMA-DPH were used to study the effects of platinum(II) complexes on the fluidity of POPC/SM/Chol (1:1:1) mixture which displays liquid-ordered/liquid-disordered phase separation and ceramide-containing mixtures with gel/fluid phase separation [POPC/C16Cer (8:2) and POPC/C24:1Cer (7:3)], where the gel phase is ceramide-enriched. A sustained decrease in membrane fluidity was detected by *t*-PnA upon adding cisplatin and AqCis to the POPC/SM/Chol mixture (Figure 4A). The effect was more pronounced in the presence of AqCis. Similarly, a transient decrease in membrane fluidity was detected by TMA-DPH upon adding AqCis to the ternary mixture (Figure 5A).

No significant differences in *t*-PnA fluorescence anisotropy were observed for the POPC/C16Cer (8:2) LUV model, with an average anisotropy over the period of 3 h of 0.2659 ± 0.0018 for control and 0.2662 ± 0.0037 and 0.2654 ± 0.0031 for 300 μM of cisplatin and AqCis, respectively (Figure 4B). However, an increase in TMA-DPH fluorescence anisotropy was observed upon addition of cisplatin (Figure 5B). At the endpoint of the experiment, the average anisotropy of TMA-DPH was 0.1785 ± 0.0012 and 0.1814 ± 0.0028 for LUV treated with 15 and 300 μM of cisplatin, respectively, and 0.1669 ± 0.0027 for the control. AqCis, on the other hand, caused a transient decrease in TMA-DPH anisotropy (0.1647 ± 0.0015 for 300 μM) compared to the control (0.1729 ± 0.0021), which was sustained at least up to 48 h It should be noted that TMA-DPH anisotropy values are typical of the fluid phase^22^ and much lower than the values measured in this study for membranes containing Lo/Ld phase separation (POPC/SM/Chol) and gel phase (DPPC). Thus, the probe is excluded from the highly ordered ceramide-enriched gel domains, and therefore, this probe solely reports changes in the fluid regions of this lipid mixture.

Finally, for POPC/C24:1Cer (7:3) LUV, transient differences in the distribution of *t*-PnA fluorescence anisotropy were observed after addition of the highest concentration of cisplatin but not AqCis (Figure 4D). Furthermore, using the Savitzky–Golay method to smooth the UV spectra data, a very small concentration-dependent effect was observed at 312 nm, 30 min after addition of cisplatin, indicating that the latter interacted with the lipid bilayer (Figure 4E). The effect of cisplatin was transient, and no differences in *t*-PnA anisotropy were found after 3 h (⟨*r⟩* of 0.2454 ± 0.0136, 0.2484 ± 0.0016, and 0.2453 ± 0.0029 for control and 300 μM of cisplatin and AqCis, respectively). These results suggest that cisplatin but not AqCis has an initial and transient effect in the packing of the lipid chains in bilayers containing C24:1Cer. However, no differences in TMA-DPH anisotropy were observed for both cisplatin and AqCis in the POPC/C24:1Cer (7:3) model (Figure 5C). While *t*-PnA incorporates the C24:1Cer gel domains, TMA-DPH is mostly excluded from these regions, as shown by the low anisotropy values obtained for this probe. Therefore, the two probes report changes induced by cisplatin in different membrane regions.

### Cisplatin-Induced Changes in Membrane Permeability

**Figure 6:**
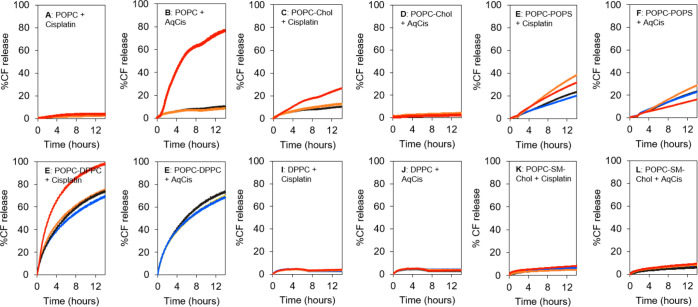
Cisplatin and AqCis-induced changes in membrane permeability. CF release from (A,B) POPC, (C,D) POPC/Chol (7:3), (E,F) POPC/POPS (7:3), (G,H) POPC/DPPC (1:1), (I,J) DPPC LUV, and (K,L) POPC/SM/Chol (1:1:1) was measured over time (up to 14 h) in the absence of platinum complexes and in the presence of (A,C,E,G,I,K) cisplatin and (B,D,F,H,J,L) AqCis, respectively, for each pair of graphs. Black: control; blue: 15 μM platinum; orange: 35 μM platinum; red: 300 μM platinum. Each time point corresponds to the average of a single read in three independent measurements.

The CF leakage assay from LUV is an established method to evaluate the membrane permeability in the presence of various molecules.^30−32^ The influence of platinum(II) complexes on the permeability of model membranes was determined by measuring the fluorescence intensity of CF upon the release of the probe from the interior of the vesicles. Figure 6A,B shows that at a therapeutic concentration of 35 μM, neither cisplatin nor AqCis induced significant effects on the release of CF from POPC vesicles. However, at a saturating concentration of 300 μM, both platinum(II) complexes increased the release of CF from these vesicles. In particular, AqCis showed a very prominent release of up to 80% of total CF (Figure 6B).

The POPC/Chol (7:3) vesicles showed a lower rate of CF release and cholesterol prevented the release of CF upon addition of high concentrations of cisplatin and AqCis. On the other hand, cisplatin and AqCis-induced release of CF from POPC/POPS (7:3) LUV changed in a nonlinear concentration-dependent manner (Figure 6E,F). At the lowest concentration of 15 μM, no differences in the release of CF were observed compared to the control. Addition of 35 μM of platinum complexes to these vesicles caused a significantly higher release of CF, particularly for those treated with cisplatin (Figure 6E). A further increase in platinum complex concentration lowered CF release from the vesicles. In particular, a lower release of CF was observed in POPC/POPS (7:3) LUV after addition of 300 μM AqCis compared to control (Figure 6F).

In model membranes containing gel phase, that is, POPC/DPPC (1:1) (Figure 6G,H) and DPPC (Figure 6I,J) LUV, no significant differences were observed for both platinum complexes except for the highest concentration of cisplatin in the POPC/DPPC (1:1) LUV, which showed to completely release the CF within the time range tested (Figure 6G). On the other hand, in the gel phase DPPC membranes, almost no release of CF was observed, with only 4–5% of CF released after 14 h for both control and platinum complexes (Figure 6I,J). It should be noted that POPC/DPPC (1:1) LUV displayed a high release of CF even in the absence of platinum complexes due to the existence of membrane packing defects caused by gel/fluid phase separation.^33^

Finally, in the POPC/SM/Chol (1:1:1) model, even though very slow release of CF was observed (Figure 6K,L), at the endpoint, it was slightly faster for both 300 μM of cisplatin (9.59%) and AqCis (10.73%) compared to control (7.57%).

## Discussion

### Effects of Cisplatin and AqCis on Fluid and Gel Phase Phospholipid Model Membranes

Drug–lipid interactions are complex, and lipid chemical structure can have a significant contribution. The impacts platinum(II) compounds have in the organization of lipid membranes, although not fully understood, include inducing lipid phase changes, mixing of plasma membrane components, and modulating the lipid metabolism and membrane composition.^14,34−36^ However, literature data regarding cisplatin–membrane interactions are controversial. For example, a study suggests that cisplatin accumulates at the headgroup region of the membrane and induces ordering of the DMPC acyl chains with a decrease in the area per lipid molecule and membrane elasticity.^19^ In contrast, another study showed no significant differences in DMPC membrane fluidity at concentrations of cisplatin many orders of magnitude higher than the therapeutic dosage (10 mM).^37^ This is in line with the results obtained for both cisplatin and AqCis on the different phospholipid model membranes tested in the present study. Our results suggest that in fluid phase model membranes, both cisplatin and AqCis have a very minor influence on the fluidity measured at the interior of the membrane, even at concentrations 10 times superior to the normal therapeutic dose. In fact, only at high concentrations, small and transient effects on lipid ordering and alterations in the permeability of POPC, POPC/DPPC, and POPC/SM/Chol membranes were observed. These effects were not attributed to the collapse of the membrane since the size of LUV remained the same as measured by DLS and seem to stem mostly from interactions at the lipid headgroups. For POPC, this effect was higher with AqCis, which suggests the involvement of the zwitterionic headgroups in the interaction, whereas for POPC/DPPC, the increased membrane permeability was only observed for cisplatin, suggesting more pronounced membrane packing defects due its incorporation at the interface between the fluid and gel phases. The previously observed small ordering of DPPC may slightly accentuate the segregation between POPC and DPPC into the fluid and the gel phases, increasing the difference in the biophysical properties of these two phases, which is sufficient to hinder even more the packing of the lipids at the gel/fluid interfaces, and resulting in higher permeability. In the latter case, the cationic species may not be favored for interactions at this interface due to the hydrophobic characteristics created by height mismatch between lipids. Previous studies have reported that platinum complexes, including cisplatin and AqCis, were able to increase the permeability of erythrocyte membranes^38^ and planar model membranes of hen’s egg yolk (mostly composed of phospholipids of PC and PE), where the latter was attributed to the formation of small holes.^39^ Our results suggest that indeed the permeability of the membrane is increased without major alterations of the lipid phase properties. Even though these observations were only at non-therapeutic concentrations, understanding the underlying mechanism is important to predict the impact of other platinum(II) species, particularly for those with a more hydrophobic character where more efficient incorporation into the membrane is expected.

Furthermore, Chol, which, due to its planar rigid structure, intercalates within the hydrocarbon chain, increases the order of the fluid phase and reduces the formation of gel phase.^40−42^ It was previously shown that digitonin, a compound that interacts with cholesterol, increased the permeability of the membrane^9^ and consequently increases cisplatin uptake.^9,13^ On the other hand, molecular dynamics simulations suggested that the addition of up to 33% of cholesterol in lipid bilayers did not influence the diffusion of cisplatin.^43^ Our results support these conclusions since no significant changes in membrane fluidity or permeability were observed upon adding cisplatin or AqCis to POPC/Chol (7:3) LUV.

Regarding platinum(II) complex interactions with gel phase phospholipid membranes, previous studies suggested that cisplatin interacts with the headgroup of DPPC forming a 2:1 lipid–platinum complex with the phosphate groups and causes rearrangements that result in lower mobility of the headgroup.^44^ AqCis was shown to strongly interact with the DPPC lipid headgroup, consistent with binding to the headgroup, and induce structural alterations of the glycerol moiety.^7,12^ This is in line with our results as it was observed that both complexes were able to induce a small but consistent concentration-dependent increase in the order of DPPC membranes. This effect was noticeable within the first minutes and was maintained throughout 72 h. Since the formation of Pt–O–P complexes was reported to be transient occurring only after 2.5 h and disappearing after 12 h,^10^ these results do not exclude that mechanisms accounting for the observed changes in DPPC membrane fluidity other than the formation of the complex may be occurring.

### Platinum(II) Complex Interactions with POPS

Phosphatidylserines are anionic phospholipids mostly located in the inner leaflet of the plasma membrane of non-cancer cells and have a crucial role in many cellular events.^4,45−47^ However, in some cancer cells, this type of lipid is increased in the outer leaflet of the plasma membrane^48,49^ and promotes changes on membrane surface charge and packing that impact chemotherapy. In particular, platinum(II) complexes were shown to specifically interact with anionic lipids including POPS, and AqCis charge may be the determining factor for the binding.^7,19,24,50,51^ Indeed, AqCis is able to increase the main transition temperature of anionic DPPG lipid but not of the zwitterionic DPPC, which was attributed to the electrostatic interactions that lead to a more rigid and less fluid membrane.^24^

Our results suggest a fast, electrostatic-driven interaction between AqCis and membranes containing the anionic lipid POPS, resulting in surface charge neutralization. From a biological perspective, this surface charge neutralization has important consequences in cells since it changes the overall surface charge potentially affecting membrane-associated events. The results further suggest that if the coordination complex was formed, it is reversed by increased chloride concentration since the zeta potential returned to values similar to the control sample. Furthermore, charge neutralization has been regarded as the mechanism by which platinum compounds rigidify the membrane, similar to effects observed for charged ions, such as Mg^2+^ and Ca^2+^ cations.^24,52^ Charge neutralization might also increase gel-to-fluid transition temperatures,^24,53^ likely due to decreased electrostatic repulsion between the lipid headgroups and a consequent increase in the lipid packing. However, no differences in the fluidity in the POPC/POPS model membranes were observed by addition of either cisplatin or AqCis, even for an extended period of time. These results suggest that both the charge neutralization and/or the potential coordination of the serine headgroup did not have a significant influence in the phase properties of this model membrane. However, a complex behavior in the permeability properties of these vesicles was observed within the first 14 h, suggesting that both cisplatin and AqCis are able to interact with POPS. As to why increasing amounts of both cisplatin and AqCis resulted in lower release of CF and in particular for the highest concentration of AqCis is yet to be determined. It is possible that cisplatin induced a more disordered membrane^18^ and in comparison to AqCis induced a higher release of CF, which could be attributed by increased defects in the membrane core that do not involve phase change.

### Effect of Platinum Complexes on Sphingolipid Mixtures

Membrane lipid domains are fundamental for many cellular signaling pathways and have already been implicated in both cisplatin-induced apoptosis^15,54,55^ and drug resistance.^56^ The so-called lipid raft domains are the primary sites for aSMase action, which is activated in response to stress stimuli, leading to the formation of ceramide-enriched domains and clustering of receptors at these lipid-based signaling platforms.^15,17,55,57^ Studies in HT29 human colon carcinoma cells showed that cisplatin-induced aSMase activation caused a transient increase in membrane fluidity for both bulk membrane and lipid rafts that led to reorganization of the lipid domains and clustering of CD95 receptors and subsequent apoptosis.^55^ However, these observations contrast with the expected alterations in membrane properties upon ceramide generation. Indeed, ceramides are highly hydrophobic and prone to form tightly packed gel phase domains, which cause a decrease in the overall fluidity of both model and biological membranes.^17,18,27,58−60^ Such evidence suggests that other factors besides ceramide formation might be responsible for the observed cisplatin-induced increase of membrane fluidity. In the present study, we showed that platinum complexes can cause a decrease in the fluidity of SM- and ceramide-containing membranes. Our data further suggest that cisplatin seems to interact with C24:1Cer gel domains causing a transient increase in their order (as measured by *t*-PnA) but not with the fluid membrane regions of this mixture (as measured by TMA-DPH). In contrast, cisplatin caused a sustained decrease in the fluidity of the fluid regions of C16Cer-containing mixtures, while no effects were observed within the gel domains. A decrease in the fluidity of POPC/SM/Chol model was observed upon interaction with AqCis and to a lower extent with cisplatin. Together, these results suggest that gel–fluid phase separation might facilitate cisplatin membrane interaction, likely due to enhanced packing defects present at the gel–fluid interface. The results could also suggest a specific interaction between cisplatin and ceramides. However, the results point to cisplatin effects occurring in membrane regions enriched in C24:1Cer but not in C16Cer. Compared to C16Cer, the gel phase formed by C24:1Cer^22^ is less rigid than the one formed by C16Cer^58^ and similar to DPPC.^61^ This might facilitate the interaction with and/or incorporation of the platinum molecules within these domains, whereas the interaction with C16Cer-containing membranes occurs mostly at the fluid phase. The presence of an unsaturation and hydrophobic chain asymmetry at the membrane interior cause chain interdigitation and might enhance packing defects that would further increase the intercalation of cisplatin and AqCis to the membrane/water interface.

Our observations show therefore that cisplatin increases the order of ceramide-containing membranes and other factors may contribute to the observed increase in fluidity at lipid rafts in HT29 cells.^15^ Interestingly, AqCis seems to cause a transient decrease in the order of the fluid regions of C16Cer-containing membranes. One possible explanation is that the targeting of packing defects at the gel–fluid interface may slow down the demixing of the lipids, that is, the attaining of the fully equilibrated situation. Overall, since cisplatin is known to easily exchange its chlorides with other biomolecules with particular high affinity for thiols,^3,7^ these could potentially interact differently with lipid bilayers and be responsible for the observations in HT29 cells.

## Conclusions

This work shows that the interaction of platinum(II) complexes with lipid membranes is complex. At therapeutic concentrations, both cisplatin and AqCis did not induce substantial changes in membrane fluidity. However, at high concentrations, these complexes seem to promote small ordering (a decrease in membrane fluidity) of membranes containing gel phase; moreover, the effects of platinum(II) complexes on membrane fluidity are lipid-type-specific and/or dependent on the characteristics of the gel phase formed. These results thus suggest that the molecular mechanism underlying the cisplatin interaction with biological membranes is not straightforward, and the effects of platinum(II) complexes in the properties of biological membranes should be further explored for other analogues.

## Supporting Information Available

Effect of cisplatin on POPC LUV; effect of cisplatin on different lipids; effect of cisplatin on POPC/POPS LUV; and effect of cisplatin on DPPC LUV (PDF)

## Abbreviations

AqCis: “Aquated” platinum species
C16Cer: C16 ceramide, *N*-palmitoyl-d-*erythro*-sphingosine
C24:1Cer: C24:1 ceramide, *N*-nervonoyl-d-*erythro*-sphingosine
CF: 5(6)-carboxyfluorescein
DPPC: 1,2-dipalmitoyl-*sn*-glycero-3-phosphocholine
POPC: 1-palmitoyl-2-oleoyl-*sn*-glycero-3-phosphocholine
SM: sphingomyelin
POPS: 1-palmitoyl-2-oleoyl-*sn*-glycero-3-phospho-*l*-serine
*t*-PnA: *trans-*parinaric acid
TMA-DPH: 1-(4-(trimethylammonium)phenyl)-6-phenylhexa-1,3,5-triene
