# Supplementary material for: Effect of Cisplatin and Its Cationic Analogues in the Phase Behavior and Permeability of Model Lipid Bilayers
Source: Mol Pharm. 2023 Jan 26;20(2):918–28. doi: 10.1021/acs.molpharmaceut.2c00321 (PMC9906771; doi:10.1021/acs.molpharmaceut.2c00321)
Supplement: Supplementary file 1 — mp2c00321_si_001.pdf [file mp2c00321_si_001.pdf]

SUPPLEMENTARY INFORMATION FOR:

Effect of cisplatin and its cationic analogues in the phase behavior and permeability of model lipid bilayers

Nuno Martinho[1,2], Joaquim M. T. Marquês[3], Iryna Todoriko[4], Manuel Prieto[2], Rodrigo F.M. de Almeida[3] and Liana C Silva[1,]*

[1] Research Institute for Medicines (iMed.ULisboa), Faculdade de Farmácia, Universidade de Lisboa. Lisboa, Portugal

[2] iBB—Institute for Bioengineering and Biosciences and Department of Bioengineering, Instituto Superior Técnico, Universidade de Lisboa, Av. Rovisco Pais, 1049-001 Lisboa, Portugal;

[3] Associate Laboratory i4HB—Institute for Health and Bioeconomy at Instituto Superior Técnico, Universidade de Lisboa, Av. Rovisco Pais, 1049-001 Lisboa, Portugal

[4] Centro de Química Estrutural, Institute of Molecular Sciences, Departamento de Química e Bioquímica, Faculdade de Ciências, Universidade de Lisboa, Campo Grande, 1749-016 Lisboa, Portugal

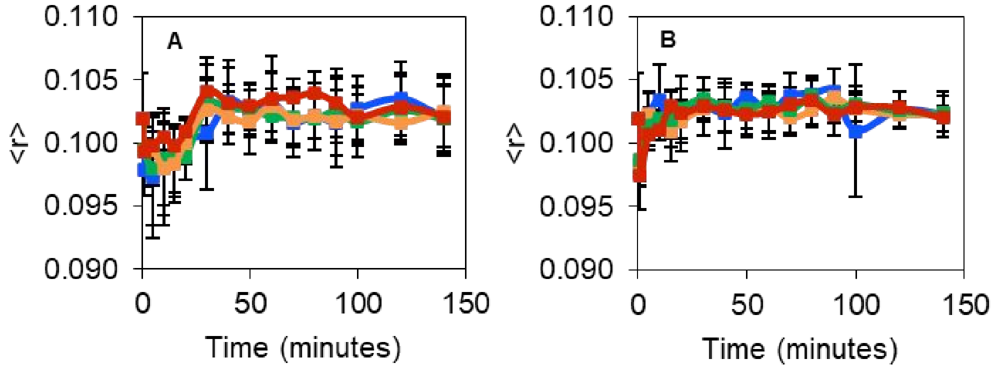

Figure S1 Cisplatin- and AqCis-induced changes on fluid POPC membranes. Time course variation of DPH fluorescence anisotropy after addition of 15 µM (blue), 35 µM (orange), 100 µM (green) and 300 µM (red) of (A) cisplatin and (B) AqCis to POPC LUVs. Data are the average ± SD of 3 independent experiments.

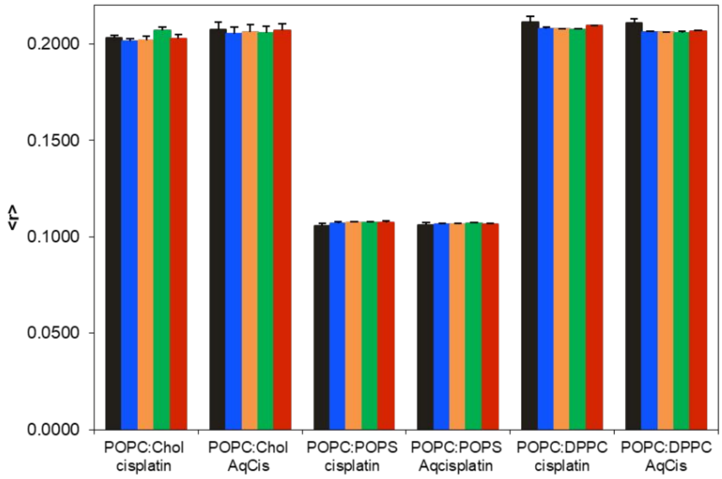

Figure S2 Effect of cisplatin and AqCis on the fluidity of membranes with different composition and membrane properties. Average fluorescence anisotropy of DPH between 30 min and 3 hours after addition of cisplatin or AqCis to LUV containing POPC:Chol (7:3), POPC:POPS (7:3), and POPC:DPPC (1:1) showed no meaningful differences compared to control. Black: control; Blue: 15 μM; Orange: 35 μM; Green: 100 μM; Red: 300 μM of cisplatin or AqCis.

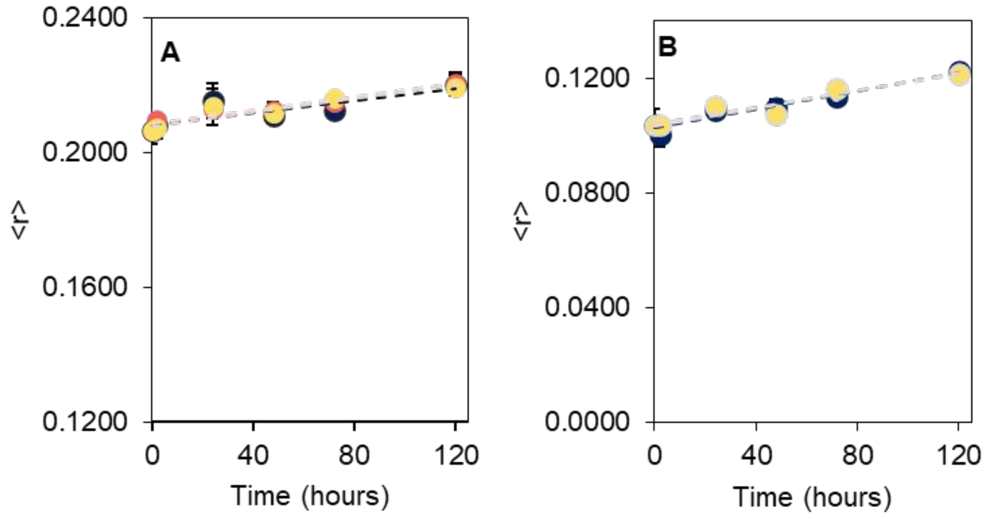

Figure S3 Effect of cisplatin and charged AqCis in the fluidity of POPC:POPS (7:3) model membranes. Fluorescence anisotropy of (A) TMA-DPH and (B) DPH in POPC:POPS (7:3) LUV in buffer B (low chloride) in the absence (black) and presence of 300 μM of cisplatin (yellow) and AqCis (pink). Data are the average ± SD of 3 independent experiments.

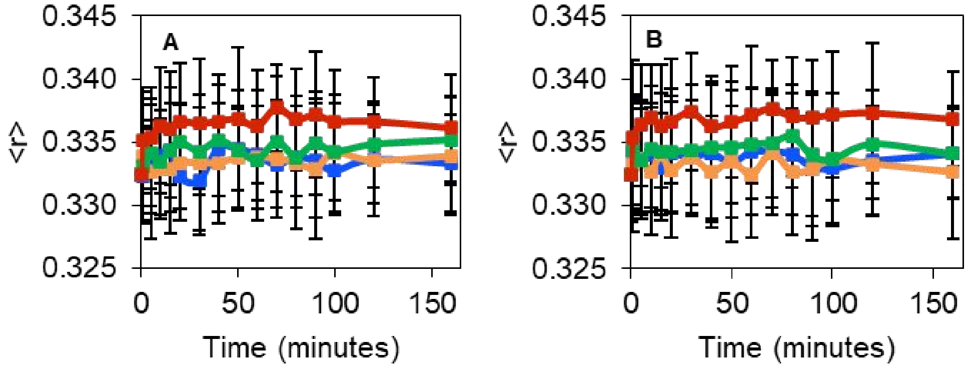

Figure S4 Cisplatin- and AqCis-induced changes on gel-phase DPPC membranes. Time course variation of DPH fluorescence anisotropy after addition of 15 μM (blue), 35 μM (orange), 100 μM (green) and 300 μM (red) of (A) cisplatin and (B) AqCis to DPPC LUVs. Data are the average ± SD of 3 independent experiments.
